# Immunotherapy choice and maintenance for generalized myasthenia gravis in China

**DOI:** 10.1111/cns.13468

**Published:** 2020-10-26

**Authors:** Chao Zhang, Bitao Bu, Huan Yang, Lihua Wang, Weibin Liu, Rui‐Sheng Duan, Meini Zhang, Pei Zeng, Chen Du, Li Yang, Fu‐Dong Shi

**Affiliations:** ^1^ Department of Neurology Tianjin Neurological Institute Tianjin Medical University General Hospital Tianjin Medical University Tianjin China; ^2^ National Clinical Research Center for Neurological Diseases of China Jing‐Jin Center for Neuroinflammation Beijing Tiantan Hospital Capital Medical University Beijing China; ^3^ Department of Neurology Beijing Tiantan Hospital Capital Medical University Beijing China; ^4^ Department of Neurology Tongji Hospital Tongji Medical College Huazhong University of Science and Technology Wuhan China; ^5^ Department of Neurology Xiangya Hospital Central South University Changsha China; ^6^ Department of Neurology The Second Affiliated Hospital Harbin Medical University Harbin China; ^7^ Department of Neurology National Key Clinical Department and Key Discipline of Neurology The First Affiliated Hospital Sun Yat‐sen University Guangzhou China; ^8^ Department of Neurology The First Affiliated Hospital of Shandong First Medical University Jinan China; ^9^ Department of Neurology The First Hospital of Shanxi Medical University Taiyuan China

**Keywords:** efficacy, real‐world, relapse, rituximab, tacrolimus

## Abstract

**Aims:**

To compare long‐term efficacy and safety of immunotherapeutic strategies as maintenance to prevent disease relapses of generalized myasthenia gravis (MG) in real‐world settings.

**Methods:**

This is a retrospective cohort study on generalized MG conducted in seven major neurological centers across China. Eligible participants were patients with generalized MG who were under minimal manifestation status or better. Main outcome measures were probability of patients free of relapses and causes of drug discontinuation.

**Results:**

Among 1064 patients enrolled, the median (interquartile range) age was 50.3 (37.0‐62.5) years and 641 (60.2%) were women. Disease relapse was significantly lower for rituximab (6.1%) compared with all the other monotherapies (hazard ratio [HR] = 0.18, 95% confidence interval [CI] 0.06 to 0.56, *P* = .0030). As combination therapies, tacrolimus in combination with corticosteroids reduced risk of disease relapses compared with azathioprine with corticosteroids (HR = 0.45, 95% CI 0.25 to 0.81, *P* = .0077) or mycophenolate mofetil with corticosteroids (HR = 0.32, 95% CI 0.15 to 0.67, *P* = .0020). Otherwise, lower‐dose corticosteroids or azathioprine as monotherapy significantly increased risk of disease relapses (HR = 2.78, 95% CI 1.94 to 3.99, *P* < .0001; HR = 2.14, 95% CI 1.42 to 3.23, *P* = .0003, respectively). The proportion of discontinuation was lowest in patients with rituximab (20.4%) as monotherapy and tacrolimus with corticosteroids (23.6%). Overall, combination treatment of immunosuppressants with corticosteroids had a lower rate of discontinuation compared with corresponding monotherapy (HR = 0.51, 95% CI 0.36 to 0.71, *P* < .0001).

**Conclusions:**

Rituximab as monotherapy and tacrolimus with corticosteroids displayed better clinical efficacy as well as drug maintenance to prevent disease relapses in patients with generalized MG.

## Graphical abstract

As monotherapy in patients with generalized myasthenia gravis, rituximab displays higher efficacy to prevent disease relapses compared to other oral immunosupressants.

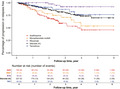

## 1 INTRODUCTION

Myasthenia gravis (MG) is an autoantibody‐mediated autoimmune disorder against postsynaptic membrane proteins at the neuromuscular junction, including acetylcholine receptor (AChR), muscle‐specific kinase (MuSK), and lipoprotein receptor‐related protein 4 (LRP4).^1^ The bulk of generalized MG therapy include acetylcholinesterase inhibitors for symptomatic management as well as immunotherapeutic agents to inhibit autoimmunity. Most patients with generalized MG require induction therapy with glucocorticosteroids (steroids), or intravenous immunoglobulin (IVIG), plasma exchange (PE) for severe cases. And immunosuppressants are frequently used for maintenance. A repertoire of classical immunosuppressants, azathioprine, cyclophosphamide, mycophenolate mofetil, and tacrolimus have also been adopted.^2^, ^3^ Some efficacy of an anti‐CD20 B‐cell depleting agent, rituximab, is demonstrated by sustained clinical improvement with prolonged time to relapse and reduce the need of other immunosuppressants.^4^, ^5^, ^6^, ^7^ Treatments with these agents are commonly initiated in conjunction with steroids so that they may be gradually tapered to a lower maintenance dose, which varies among individual patients.^8^ A previous network meta‐analysis showed the efficacy and tolerability of the immunosuppressants were heterogeneous.^9^

Presently, no consensus has been reached on an optimal therapeutic maintenance protocol in the prevention of relapses in generalized MG.^10^ Using the MG registry of seven major centers throughout China, we analyzed the safety and efficacy of steroids and nonsteroidal immunotherapeutic agents in MG patients who had achieved Minimal Manifestation Status (MMS) or better during the long‐term follow‐up.

## 2 METHODS

### 2.1 Study design and patients

This retrospective cohort study was based on the registry databases established in 7 independent neurological centers across China. Patient data were collected from August 9, 2013, to September 30, 2019, and data were censored thereafter. Institutional review boards approved this study at each participating center, and informed consent was obtained from each patient.

Generalized MG was diagnosed by a consulting neurologist, based on compatible clinical features together with one or more of the following criteria: (a) seropositivity in anti‐AChR, MuSK, or LRP4 antibody assay; (b) electrophysiological study findings compatible with a postsynaptic neuromuscular junction disorder (repetitive stimulation, single‐fiber electromyography, or both); and (c) a response to cholinesterase inhibitors. In cases of seronegativity, MG diagnosis was confirmed by abnormal findings from neurophysiological studies.

**Figure 1:**
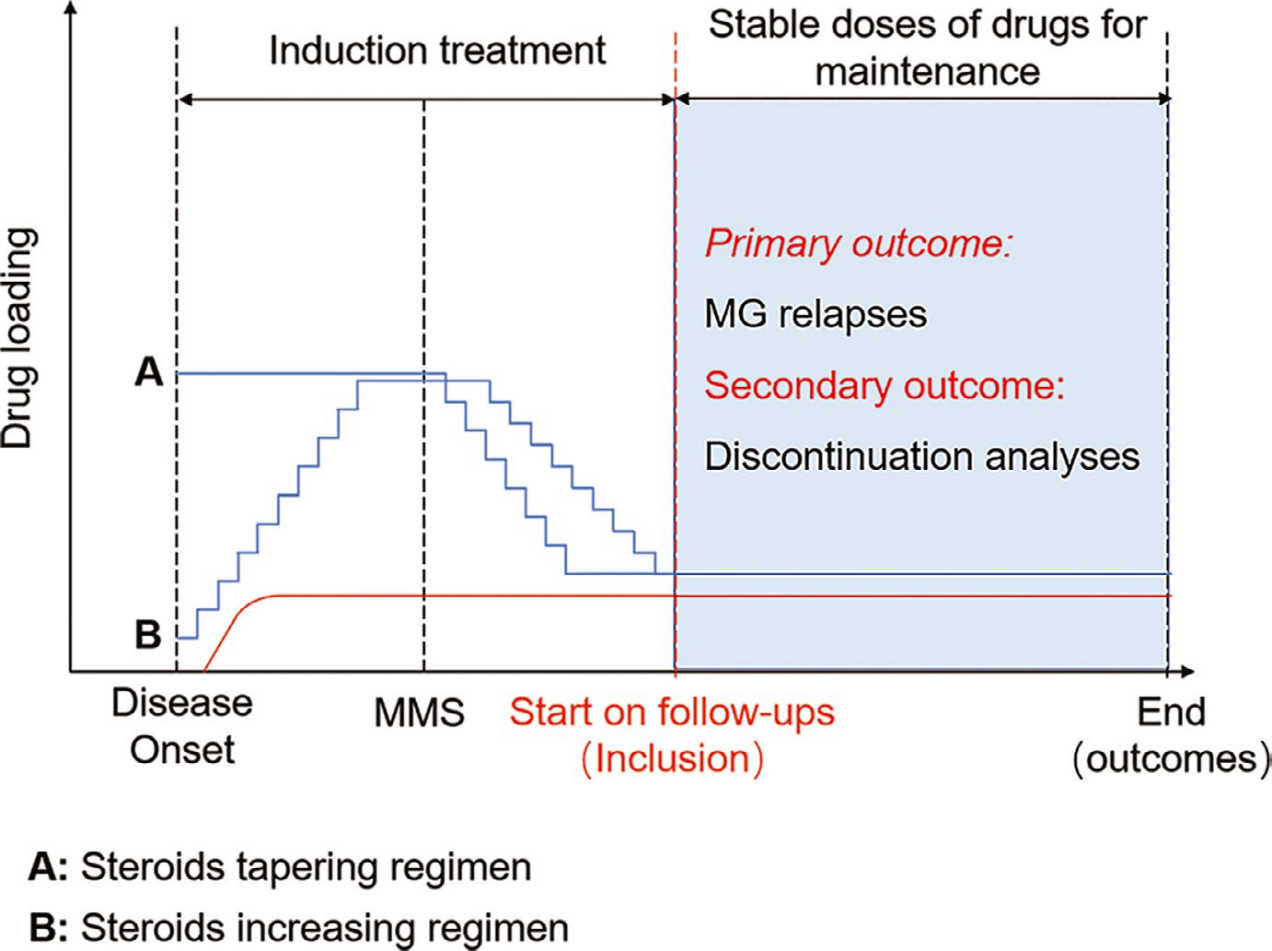
Treatment Strategies Before and After Study Inclusion. At disease onset, there were 2 protocols of corticosteroids usage to achieve remission or MMS. Patients received corticosteroids at high doses (1‐1.5 mg/kg/d) and maintained 1‐3 months to improve symptoms. The other patients resumed gradual corticosteroids escalation, starting with a dose of 10‐20 mg/d and increased by 10 mg per week until the main symptoms improved. Then corticosteroids tapered. Immunosuppressants that aimed to reduce corticosteroids were added before MMS until stable doses were attained. Once the patients met criteria of inclusion, corticosteroids and immunosuppressants were used alone or in combination. The follow‐up was ended when the patients discontinued stable maintenance treatments due to disease relapses, adverse events or pregnancy

Inclusion criteria were as follows: (a) age ≥ 18 years; (b) patients who had received prior efficacious treatments and achieved treatment goals, that is, MMS or better, classified by MGFA Task Force postintervention status (PIS); patient presents no symptoms or functional limitations from MG but has some weakness on examination of some muscles; (c) steroids were used alone or in conjunction with a nonsteroidal immunotherapeutic agent, and these regimens were maintained at a constant dose (Figure 1).

Stable doses of different immunosuppressants were as follows: azathioprine 2‐3 mg/kg/d, tacrolimus 2‐5 mg/d, and mycophenolate mofetil 1‐3 g/d. Lower‐dose steroids was defined as <0.25 mg/kg prednisone or equivalent prednisolone/methylprednisolone daily.^11^ Combined regimens were defined by combination of lower‐dose steroids and an immunosuppressant at the aforementioned dosages. Higher‐dose steroids were defined as ≥0.25 mg/kg prednisone or equivalent prednisolone/methylprednisolone daily. Rituximab was administered to reduce the frequency of CD19^+^ B cells to less than 1% in peripheral blood mononuclear cells, as quantified by flow cytometry.^12^ If steroids were contraindicated or refused, a singular nonsteroidal immunotherapeutic agent was used instead. In such circumstances, early combination with steroids could be withdrawn only after the nonsteroidal immunotherapeutic agent had reached its maximal effects without disease progression or relapse.

Exclusion criteria were defined: (a) patients who participated in randomized clinical trials with unknown treatment allocation; (b) lack of follow‐up data; (c) patients who did well on immunotherapeutic treatments and discontinued treatments on their own. Patients were censored at treatment discontinuation regarding drug survival.

### 2.2 Diagnosis of relapses and refractory MG

Disease relapses, MGC, discontinuation or switching and stated reasons, and adverse events were recorded. Relapse of MG was defined when a 3‐point change or more was identified in MG‐Composite (MGC) quantitative measure.^13^ Refractory MG was defined as that postintervention status remained unchanged or worse after corticosteroids and at least 2 other immunosuppressant agents, used in adequate doses for an adequate duration, with persistent symptoms or side effects that limit functioning.^14^

### 2.3 Data collection and processing

The main clinical features of the disease were recorded: the subtypes of generalized MG onset according to the Myasthenia Gravis Foundation of America (MGFA) clinical classification^15^; presence of thymoma by thoracic computed tomography or thymus pathology in patients undergoing thymectomy; and treatment required (no treatment or cholinesterase inhibitors, and immunosuppressive therapy). Demographic data (sex, date of birth, age at onset) as well as outcomes of assays for anti‐AChR, MuSK, and LRP4 were also recorded. Testing for low‐affinity anti‐AChR antibody was not available. Upon onset of generalized MG, all patients received induction treatments with high‐dose steroids, and/or IVIG, PE to reach MMS status, which was maintained by steroids and/or immunosuppressants to prevent relapses. Steroids were tapered to stable doses for concomitant immunosuppressants or withdrawn after immunosuppressants started to exert an apparent maximal effect, based on clinicians’ experience.

Adverse events were graded according to the National Cancer Institute's Common Terminology Criteria for Adverse Events (CTCAE 5.0).

Primary outcome was the probability of patients free of relapses. The baseline time was when the patients had MMS or better and concomitantly received stable dosage of various regimens. Secondary outcomes included discontinuation of therapy, for any reason including disease relapses, pregnancy, and adverse events. All outcomes were prespecified prior to data analysis. The assessment of efficacy of immunotherapeutic agents as monotherapy commenced upon treatment duration without concomitant steroids.

### 2.4 Statistical analyses

Descriptive statistics of study cohort was performed. Continuous variables were expressed with median (IQR). Fisher's exact test was adopted to compare the categorical variables of age, gender, and previous MGFA subtype. Shapiro‐Wilk method was used for normality test. Data that did not exhibit a normal/Gaussian distribution were analyzed via the nonparametric Kruskal‐Wallis test. Kaplan‐Meier curves and Cox proportional hazards models were used to visualize and compare the probability of disease relapse and drug survival over timescale from first drug administration to outcome of interest endpoint date. Potential confounding variables: age, sex, historical MGFA subtypes, serostatus of auto‐antibodies and follow‐up time were examined and adjusted via sequential regression models. Cumulative incidences were estimated to compare the efficacy and rationale for therapy discontinuation over time between drug categories. To compare predictors between different treatments, we performed post hoc analyses for each treatment subgroup. Mixed effect Cox proportional hazards model was used to adjust the confounding factors, and centers were adjusted as a random effect factor. Statistical analyses and data processing were performed in R, version 3.4.0 (R Foundation) with the survival (version 2.41‐3), cmprsk (version 2.2‐7), ggplot2 (version 2.2.1), and survminer packages. Significance was set at a *P* value of less than .05.

## 3 RESULTS

### 3.1 Study population

**Figure 2:**
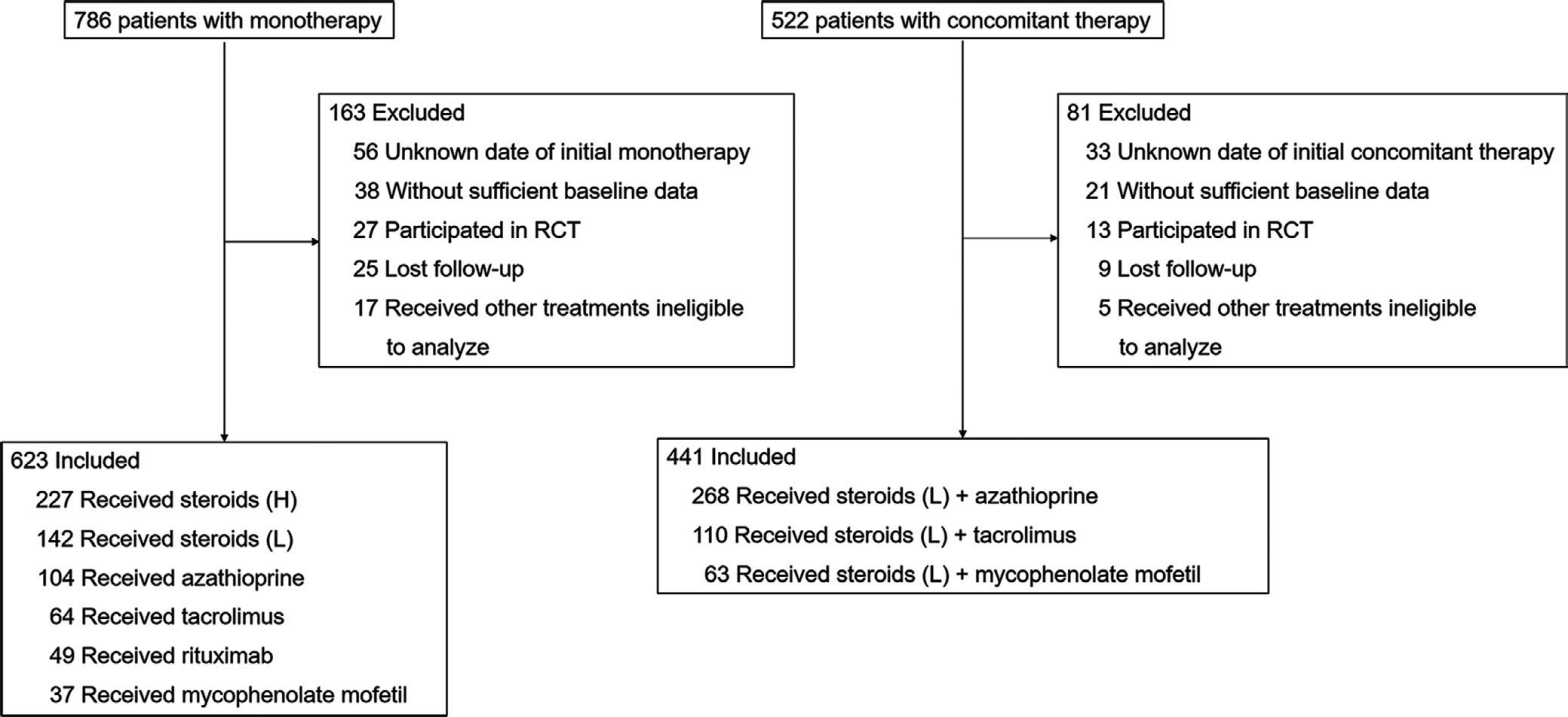
Cohort Inclusions and Exclusions for Treatment Groups. The patients were retrospectively enrolled between August 9, 2013, and September 30, 2019. The patients initially achieved the status of minimal manifestations and began stable dosing of steroids or concomitant immunosuppressants in 7 centers. RCT, randomized clinical trial

244 patients were excluded for not meeting the inclusion criteria. The study cohort comprised of 1064 generalized MG patients, of which 641 (60.2%) were women, and allocated to nine different treatments (Figure 2). The distribution of the patients at each center was as follows: 521 (49.9%), 195 (18.3%), 119 (11.2%), 102 (9.6%), 75 (7.0%), 22 (2.1%), and 20 (1.9%). Baseline characteristics for all groups are listed in Table 1. 623 patients (58.6%) received monotherapy, and remaining others (41.4%) received combined therapy as long‐term maintenance. The median (interquartile range) age of disease onset was 50.3 (37.0‐62.5) years; 50.8% of all patients were late‐onset (≥50 years). The percentage of patients with anti‐AChR antibody positivity, anti‐MuSK antibody positivity, and anti‐LRP4 antibody positivity were 65.6%, 3.2%, and 1.9%, respectively. 28.2% of the patients had thymoma, confirmed by pathology after thymectomy. The median duration of maintenance treatment was 2.3 (1.2‐3.7) years. The treatments did not differ significantly concerning sex and proportion of patients positive for anti‐AChR antibody (Table 1). There were no differences in the protocol and execution of follow‐ups between centers and treatments.

**Table 1:** Patient baseline characteristics

Treatment group									
Subtypes	Steroids (L) (n = 142)	Steroids (H) (n = 227)	AZA (n = 104)	MMF (n = 37)	TAC (n = 64)	RTX (n = 49)	Steroids (L)+AZA (n = 268)	Steroids (L)+MMF (n = 63)	Steroids (L)+TAC (n = 110)
Age, yr, median (IQR)	57.5 (43.8‐66.0)	48.3 (37.2‐60.1)	46.9 (34.7‐61.3)	54.0 (45.0‐63.7)	54.4 (42.9‐ 63.1)	59.3 (44.3‐69.5)	47.0 (32.2‐57.0)	51.9 (36.3‐64.1)	47.6 (36.3‐60.5)
Sex, Female, No. (%)	96 (67.6)	131 (57.7)	68 (65.4)	26 (70.3)	36 (56.3)	27 (55.1)	159 (59.3)	32 (50.8)	66 (60.0)
*Clinical presentation*									
Early onset (<50 years), No. (%)	50 (35.2)	123 (54.2)	56 (53.8)	14 (37.8)	26 (40.6)	20 (40.8)	150 (56.0)	29 (46.0)	56 (50.9)
Late onset (≥50 years), No. (%)	92 (64.8)	104 (45.8)	48 (46.2)	23 (62.2)	38 (59.4)	29 (59.2)	118 (44.0)	34 (54.0)	54 (49.1)
*Classified by antibodies*									
Anti‐AChR, No. (%)	116 (81.7)	183 (80.6)	78 (75.0)	32 (86.5)	53 (82.8)	43 (87.8)	194 (72.4)	41 (65.1)	89 (80.9)
Anti‐MuSK, No. (%)	3 (2.1)	9 (4.0)	3 (2.9)	1 (2.7)	3 (4.7)	4 (8.2)	8 (3.0)	3 (4.8)	4 (3.6)
Anti‐LRP4, No. (%)	1 (0.7)	3 (1.3)	5 (4.8)	0 (0)	1 (1.6)	0 (0)	3 (1.1)	2 (3.2)	1 (0.9)
Seronegative, No. (%)	11 (7.7)	16 (7.0)	11 (10.6)	4 (10.8)	2 (3.1)	2 (4.1)	22 (8.2)	7 (11.1)	6 (5.5)
NA	11 (7.7)	16 (7.0)	7 (6.7)	0 (0)	5 (7.8)	0 (0)	41 (15.3)	10 (15.9)	10 (9.1)
*Disease onset MGFA subtype*									
IIa	49 (34.8)	104 (46.0)	43 (41.3)	21 (56.8)	19 (29.7)	18 (36.7.1)	89 (33.2)	22 (34.9)	31 (28.2)
IIb	49 (34.8)	74 (32.7)	33 (31.7)	11 (29.7)	21 (32.8)	20 (40.8)	111 (41.4)	29 (46.0)	47 (42.7)

Treatment group									
Subtypes	Steroids (L)	Steroids (H)	AZA	MMF	TAC	RTX	Steroids (L)+AZA	Steroids (L)+MMF	Steroids (L)+TAC
IIIa	20 (14.2)	19 (8.4)	15 (14.4)	5 (13.5)	10 (15.6)	3 (6.1)	29 (10.8)	2 (3.2)	12 (10.9)
IIIb	12 (8.5)	22 (9.7)	5 (4.8)	0 (0)	6 (9.4)	4 (8.2)	18 (6.7)	2 (3.2)	9 (8.2)
IVa	7 (5.0)	3 (1.3)	2 (1.9)	0 (0)	4 (6.3)	2 4.1)	2 (0.7)	3 (4.8)	2 (1.8)
IVb	2 (1.40)	1 (0.40)	3 (2.9)	0 (0)	3 (4.7)	1 (2.0)	5 (1.9)	3 (4.8)	2 (1.8)
V	2 (1.40)	3 (1.30)	3 (2.9)	0 (0)	1 (1.6)	1 (2.0)	14 (5.2)	2 (3.2)	7 (6.4)
From disease onset to study inclusion (months)	9.3 (7.2‐11.5)	8.1 (6.9‐9.3)	10.2 (8.9‐11.6)	9.4 (8.3‐10.5)	8.5 (6.8‐10.3)	5.9 (4.8‐7.0)	8.3 (6.8‐9.8)	7.4 (5.8‐9.1)	7.2 (6.1‐8.4)
Thymoma, No. (%)	37 (26.1)	53 (23.3)	33 (31.7)	12 (32.4)	15 (23.4)	32 (65.3)	64 (23.9)	23 (36.5)	31 (28.2)
*Concomitant diseases*									
Autoimmune diseases	12 (8.5)	15 (6.6)	9 (8.6)	3 (8.1)	6 (9.4)	8 (16.3)	26 (9.7)	11 (17.5)	12 (10.9)
Diabetes mellitus	15 (10.1)	25 (11.0)	5 (4.8)	4 (10.8)	6 (9.4)	6 (12.2)	30 (11.2)	6 (9.5)	3 (2.7)
Hypertension	27 (19.0)	21 (9.3)	4 (3.8)	2 (5.4)	7 (10.9)	4 (8.2)	30 (11.2)	12 (19.0)	17 (15.5)
From disease onset to study inclusion (months)	9.3 (7.2‐11.5)	8.1 (6.9‐9.3)	10.2 (8.9‐11.6)	9.4 (8.3‐10.5)	8.5 (6.8‐10.3)	5.9 (4.8‐7.0)	8.3 (6.8‐9.8)	7.4 (5.8‐9.1)	7.2 (6.1‐8.4)
Follow‐up time, yr, median (IQR)	1.5 (0.5‐2.7)	2.4 (1.1‐3.7)	2.0 (1.3‐2.7)	2.5 (1.7‐3.9)	1.6 (1.2‐3.4)	3.1 (2.2‐3.9)	2.5 (1.1‐3.9)	2.4 (1.3‐3.5)	3.1 (2.0‐4.3)

Abbreviations: AChR, acetylcholine receptor; AZA, azathioprine; IQR, interquartile range; LRP4, lipoprotein receptor‐related protein 4; MGFA, Myasthenia Gravis Foundation of America; MMF, mycophenolate mofetil; MMS, minimal manifestation status; MuSK, muscle‐specific kinase; NA, not available; RTX, rituximab; Steroids (L)+AZA, lower‐dose steroids with azathioprine (steroid‐sparing azathioprine); Steroids (L)+MMF, lower‐dose steroids with mycophenolate mofetil (steroid‐sparing mycophenolate mofetil); Steroids (L)+TAC, lower‐dose steroids with tacrolimus (steroid‐sparing tacrolimus); TAC, tacrolimus.

### 3.2 MG relapses

256 patients (24.1%) experienced relapses during the documented treatments. Lower‐dose steroids, in combination with azathioprine, occupy the first‐line treatment option and encompass the largest number group of patients; we prespecified this group as the reference group for comparative analysis of efficacy and drug usage. Efficacy following adjustments for the confounding variables of age, gender, and historical MGFA subtypes, autoantibody serostatus revealed similar results to the crude analyses among the subgroups compared with the reference group (Table 2).

**Table 2:** Outcomes for treatment groups

Treatment groups									
	Steroids (L)+AZA	Steroids (L)+MMF	Steroids (L)+TAC	Steroids (L)	Steroids (H)	AZA	MMF	TAC	RTX
*Clinical relapses*									
Patients with relapses, No. (%)	60 (22.4)	17 (27.0)	14 (12.7)	63 (44.4)	39 (17.2)	39 (37.5)	13 (35.1)	8 (12.5)	3 (6.1)
Person‐years	693.78	160.72	343.86	273.53	546.85	223.31	101.78	136.88	149.65
HR, crude (95% CI)	‐	1.22 (0.71‐2.09)	0.47 (0.26‐0.85)	2.69 (1.89‐3.84)	0.83 (0.56‐1.24)	2.05 (1.37‐3.08)	1.49 (0.82‐2.72)	0.68 (0.33‐1.43)	0.23 (0.07‐0.74)
*P* value		.4741	.0114	<.0001	.3662	.0005	.1910	.3113	.0137
HR, adjusted (95% CI)	‐	1.19 (0.69‐2.05)	0.45 (0.25‐0.81)	2.78 (1.94‐3.99)	0.85 (0.57‐1.28)	2.14 (1.42‐3.23)	1.75 (0.95‐3.23)	0.69 (0.33‐1.45)	0.27 (0.08‐0.86)
*P* value		.5297	.0077*	<.0001*	.4390	.0003*	.0745	.3265	.0268*
*Thymoma (A, AB, B1 type) and no thymoma*									
Patients with relapses, No. (%)	30 (18.2)	12 (25.5)	10 (11.5)	44 (45.8)	32 (17.5)	32 (39.0)	8 (28.6)	6 (12.5)	1 (3.3)
HR, crude (95% CI)	‐	1.29 (0.66‐2.52)	0.51 (0.25‐1.04)	3.47 (2.18‐5.53)	1.00 (0.61‐1.65)	2.46 (1.49‐4.05)	1.31 (0.60‐2.85)	0.84 (0.35‐2.01)	0.16 (0.02‐1.15)
*P* value		.4576	.0631	<.0001	.9989	.0004	.5033	.6887	.0689
HR, adjusted (95% CI)	‐	1.25 (0.63‐2.46)	0.50 (0.24‐1.02)	3.55 (2.22‐5.67)	0.99 (0.60‐1.64)	2.48 (1.49‐4.10)	1.44 (0.65‐3.17)	0.80 (0.33‐1.95)	0.17 (0.02‐1.24)
*P* value		.5205	.0573	<.0001*	.9771	.0004*	.3705	.6269	.0806
*Thymoma (B2, B3, C type)*									
Patients with relapses, No. (%)	30 (29.1)	5 (31.2)	4 (17.4)	19 (41.3)	7 (15.9)	7 (31.8)	5 (55.6)	2 (12.5)	2 (10.5)
HR Crude (95% CI)	‐	1.46 (0.56‐3.79)	0.52 (0.18‐1.49)	1.85 (1.04‐3.30)	0.65 (0.28‐1.47)	1.68 (0.73‐3.87)	3.04 (1.17‐7.90)	0.52 (0.13‐2.20)	0.31 (0.07‐1.29)
*P* value		.4344	.2245	.0361	.2975	.2209	.0224	.3772	.1073
HR adjusted (95% CI)	‐	1.60 (0.60‐4.22)	0.44 (0.15‐1.27)	1.93 (1.05‐3.54)	0.71 (0.30‐1.64)	1.99 (0.85‐4.66)	4.32 (1.58‐11.80)	0.67 (0.16‐2.87)	0.42 (0.10‐1.84)
*P* value		.3472	.1272	.0347*	.4162	.1137	.0044*	.5929	.2499
*Drug discontinuation*									
Patients who discontinued therapy, No. (%)	89 (33.2)	31 (49.2)	26 (23.6)	79 (55.6)	108 (47.6)	56 (54.8)	22 (59.5)	24 (37.5)	10 (20.4)
Median drug survival time, yr	5.41	3.50	3.13	2.27	3.87	2.55	3.55	3.82	5.19
HR, crude (95% CI)	‐	1.51 (1.01‐2.28)	0.58 (0.37‐0.89)	2.27 (1.67‐3.08)	1.42 (1.07‐1.90)	2.20 (1.57‐3.06)	1.73 (1.08‐2.75)	1.45 (0.92‐2.27)	0.53 (0.28‐1.02)
*P* value		.0467	.0134	<.0001	.0164	<.0001	.0221	.1094	.0579
HR, adjusted (95% CI)	‐	1.53 (1.01‐2.31)	0.56 (0.36‐0.87)	2.27 (1.69‐3.13)	1.44 (1.08‐1.93)	2.25 (1.60‐3.14)	1.91 (1.19‐3.08)	1.48 (0.94‐2.33)	0.58 (0.30‐1.13)
*P* value		.0446*	.0101*	<.0001*	.0145*	<.0001*	.0079*	.0950	.1105

Abbreviations: AZA, azathioprine; HR, Hazard Ratio; MMF, mycophenolate mofetil; RTX, rituximab; TAC, tacrolimus.

* Indicates significance (*P* < .05) compared to patients on lower‐dose steroids in combination with azathioprine.

**Figure 3:**
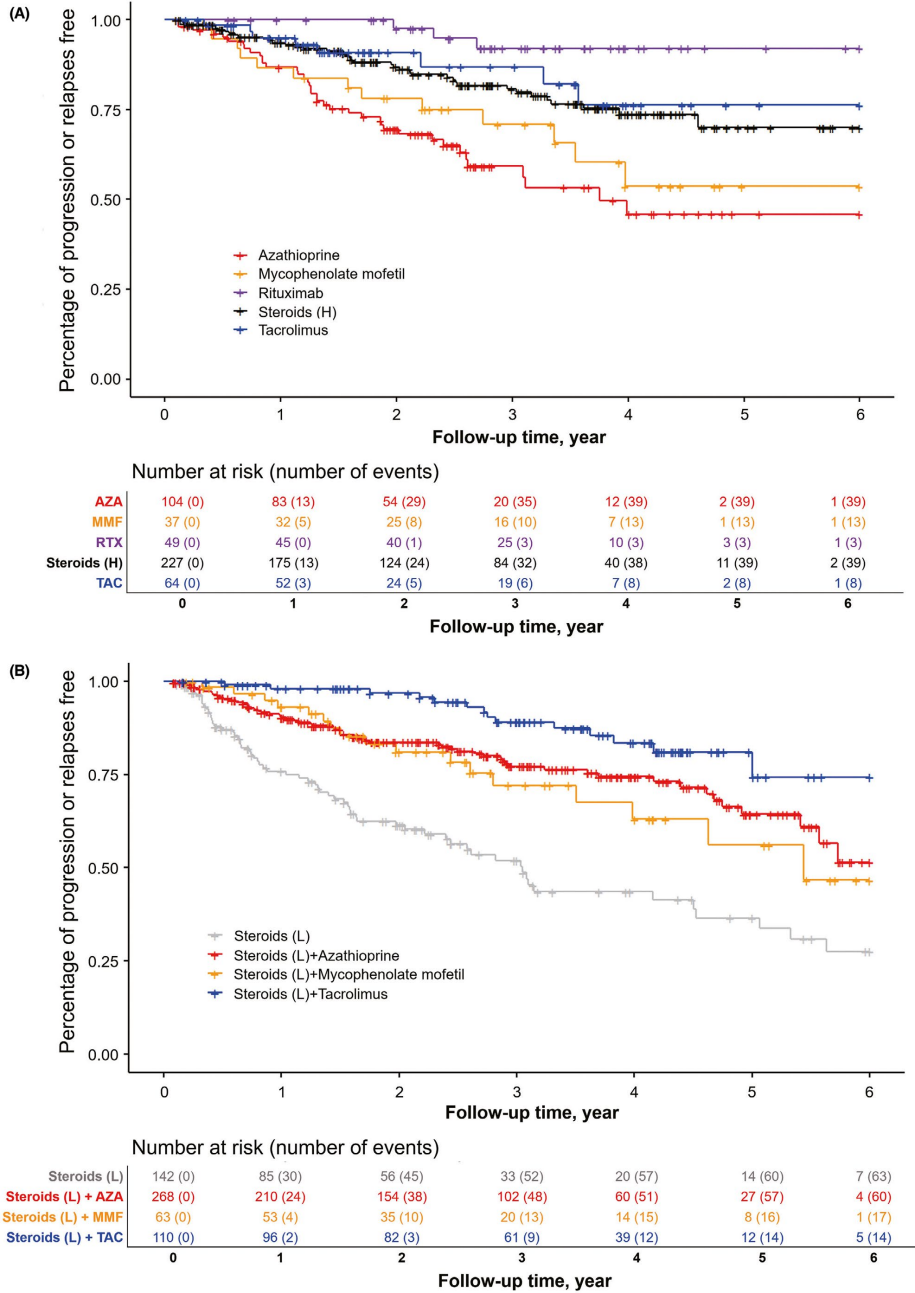
Outcomes of Disease Relapse with Different Treatments Alternatives in Patients with Generalized Myasthenia Gravis. Kaplan‐Meier curve for cumulative incidence of disease relapse for treatment groups as initial monotherapy (A) and as initial concomitant therapy, compared with lower‐dose steroids treatment (B). Rituximab reduced risk of disease relapse significantly as monotherapy. Tacrolimus in conjunction with lower‐dose steroids lowered risk of disease relapse compared to azathioprine with lower‐dose steroids or mycophenolate mofetil with lower‐dose steroids. Most relapses occurred in patients on lower‐dose steroids. AZA, azathioprine; MMF, mycophenolate mofetil; TAC, tacrolimus; RTX, rituximab

In our analysis restricted to monotherapies, disease relapse was markedly lower in rituximab‐treated patients (6.1%) compared with other immunotherapeutic agent groups in the Cox proportional hazards model (hazard ratio [HR] = 0.18, 95% confidence interval [CI] 0.06 to 0.56, *P* = .0030). Conversely, lower‐dose steroids recorded the highest risk of relapses compared with the reference group (HR = 2.78, 95% CI 1.94 to 3.99, *P* < .0001). No differences in patients receiving higher‐dose steroids or tacrolimus compared with the reference group were detected (*P* = .4390 or *P* = .3265, respectively). However, patients treated with azathioprine recorded a higher risk for relapse (HR = 2.14, 95% CI 1.42 to 3.23, *P* = .0003). Post hoc analysis showed that there was no difference between azathioprine and mycophenolate mofetil efficacy (HR = 1.32, 95% CI 0.73 to 2.40, *P* = .3909). Tacrolimus reduced relapse risk when compared with azathioprine (HR = 0.41, 95% CI 0.23 to 0.75, *P = *.0058) but not mycophenolate mofetil (HR = 0.45, 95% CI 0.19 to 1.07, *P* = .0814; Table 2 and Figure 3A).

As combined therapy, tacrolimus with lower‐dose steroids had lower risks of relapses than azathioprine with lower‐dose steroids or mycophenolate mofetil with lower‐dose steroids (HR = 0.45, 95% CI 0.25 to 0.81, *P* = .0077; HR = 0.32, 95% CI 0.15 to 0.69, *P* = .0020, respectively). There was no significant difference in risk reduction between azathioprine with lower‐dose steroids and mycophenolate mofetil with lower‐dose steroids (HR = 1.19, 95% CI 0.69 to 2.05, *P* = .5297; Figure 3B).

### 3.3 Different treatment response classified by serostatus

Anti‐MuSK positive patients had a higher risk of relapse (52.6%) than the anti‐AChR sero‐positive cohort (21.4%) (HR = 2.75, 95% CI 1.72 to 4.39, *P* < .0001); moreover, they also had a poor response to drug maintenance (34.2%) compared to anti‐AChR positive patients (60.2%) (HR = 1.94, 95% CI 1.25 to 2.85, *P* = .0024). We identified no significant differences in disease relapse or drug discontinuation between anti‐AChR positive and seronegative cohorts.

### 3.4 Predictors of relapse risk

The analysis for independent treatment response predictors revealed the risk of relapses was not associated with historical MGFA subtypes. Age of disease onset was not significantly associated with relapse risk. Adjusted for serostatus, age, disease duration, and historical MGFA types, only rituximab (HR = 0.32, 95% CI 0.10 to 0.98, *P* = .0489) and tacrolimus in combination with steroids (HR = 0.49, 95% CI 0.24 to 0.99, *P* = .0464) reduced the attack risk compared with the reference therapy. For concomitant diseases with generalized MG, autoimmune diseases, including connective tissue diseases, autoimmune thyroid disorders, were predictive for relapses (HR = 1.88, 95% CI 1.24 to 2.85, *P* = .0031). Neither diabetes mellitus nor gender was associated with higher relapse risk (Figure S1).

### 3.5 Association of relapses with thymoma types under different treatments

The efficacy of study regimens in generalized MG patients with thymoma was also analyzed. B2/B3/C thymoma types are more aggressive and an independent risk factor for postoperative myasthenic progression or crisis in MG patients. Therefore, they were separately grouped from patients with A/AB/B1 thymoma types and without thymoma. Adjusted risk analysis showed that lower‐dose steroids were least effective in preventing relapses in both subgroups (HR = 3.55, 95% CI 2.22 to 5.67, *P* < .0001; HR = 1.93, 95% CI 1.05 to 3.54, *P* = .0347). Likewise, monotherapy of azathioprine did not reduce the risk of relapses in the subgroup of patients with A/AB/B1 thymoma types and without thymoma (HR = 2.48, 95% CI 1.49 to 4.10, *P* = .0004). Mycophenolate mofetil did not reduce the risk in the subgroup of patients with B2/B3/C thymoma groups, as compared with the reference group (HR = 4.32, 95% CI 1.58 to 11.80, *P* = .0044; Table 2). However, similar efficacy was seen in the treatment groups of combined therapies as well as rituximab as monotherapy, irrespective of thymoma subtype.

### 3.6 Drug discontinuation

**Figure 4:**
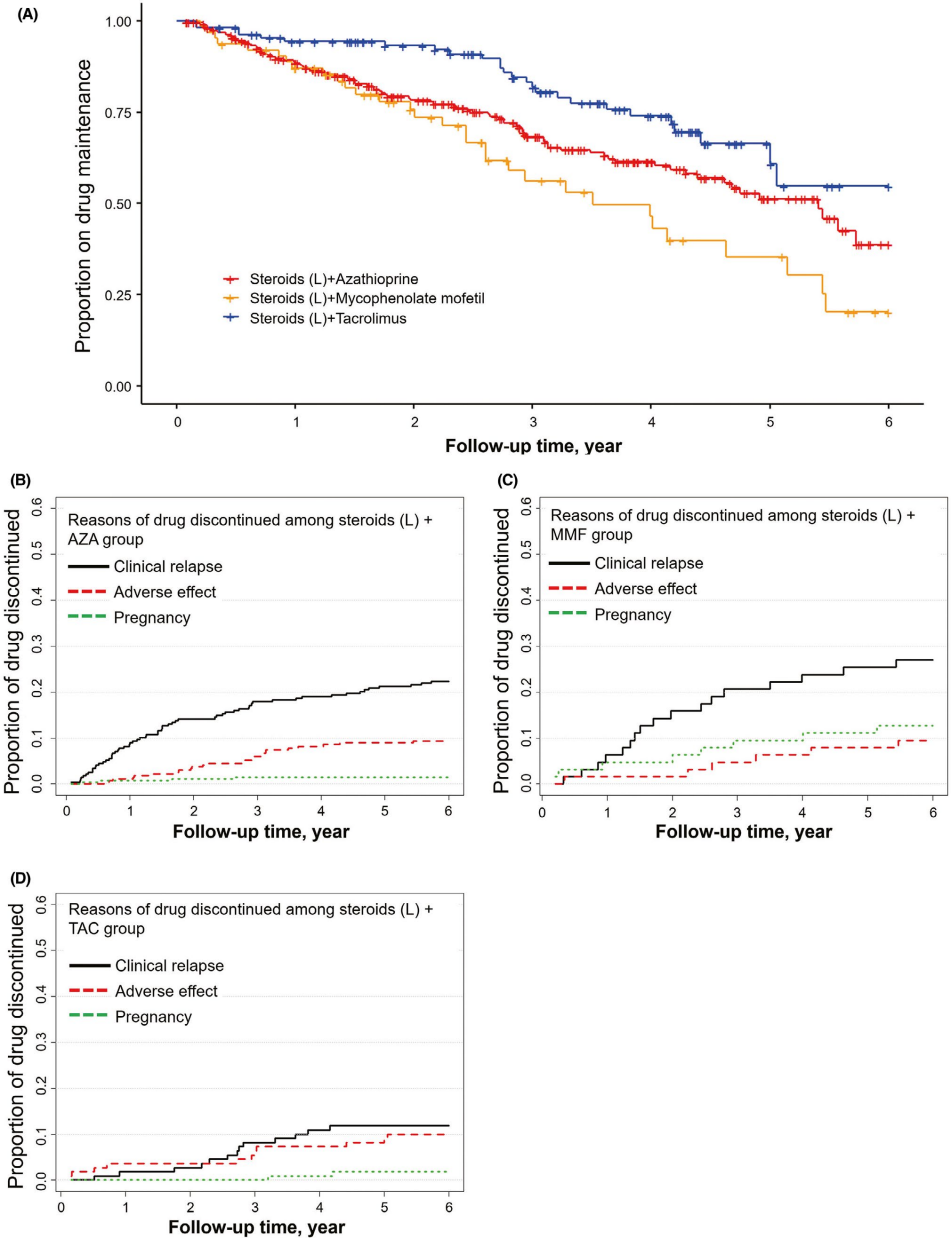
Drug Survival and Reasons for Therapy Discontinuation for Treatment Groups as combined Therapy. Comparison of different regimens for drug survival (A) and cumulative incidence reasons for therapy discontinuation for azathioprine with lower‐dose steroids (B), mycophenolate mofetil with lower‐dose steroids (C), and tacrolimus with lower‐dose steroids (D). The most common reason for these three groups was disease relapse. AZA, azathioprine; MMF, mycophenolate mofetil; TAC, tacrolimus

**Figure 5:**
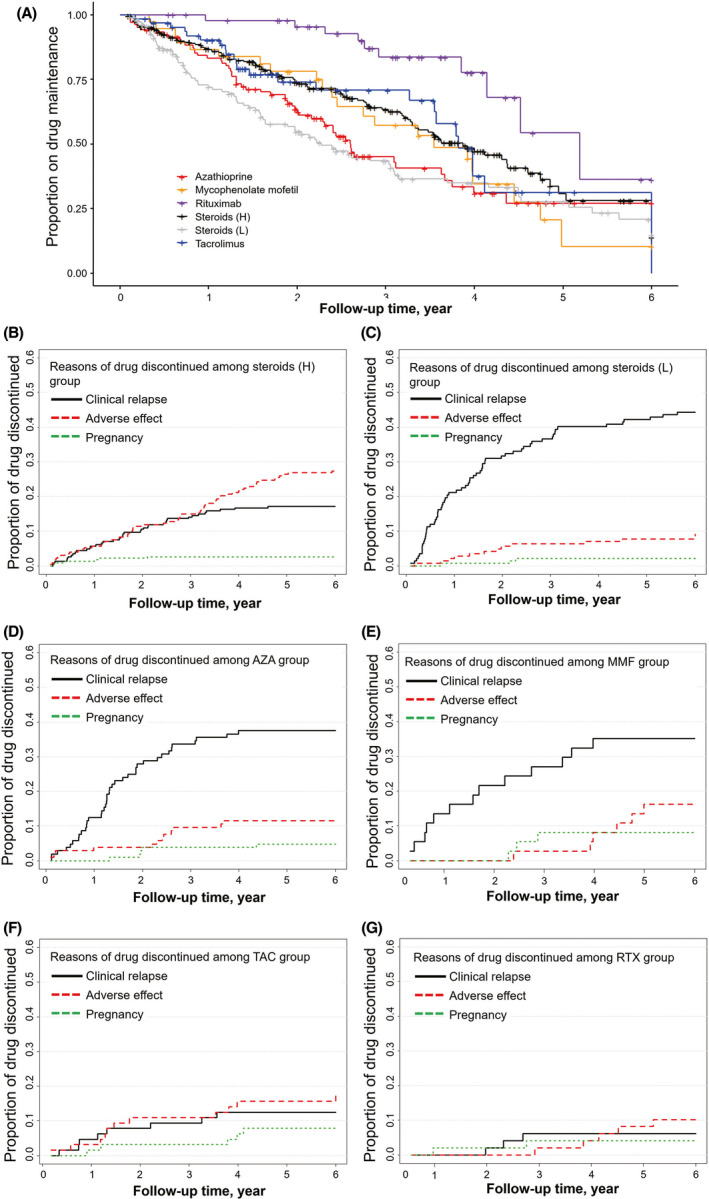
Drug Survival and Reasons for Therapy Discontinuation for Treatment Groups as monotherapy. Comparison of several immunosuppressants for drug survival (A), cumulative incidence reasons for therapy discontinuation of higher‐dose steroids (B), lower‐dose steroids (C), azathioprine (D), mycophenolate mofetil (E), tacrolimus (F), and rituximab (G). The most frequent reason for treatment stoppage of lower‐dose steroids, azathioprine, and mycophenolate mofetil was disease breakthrough; the most common reason for cessation of tacrolimus and rituximab was adverse events. AZA, azathioprine; MMF, mycophenolate mofetil; TAC, tacrolimus; RTX, rituximab

The 873 (82.1%) of all patients maintained the treatments in our cohorts. The median drug survival time varied among the regimens. Patients who received rituximab or tacrolimus with steroids had the longest drug maintenance of over 3 years. Overall, combined treatments reduced risk of discontinuation compared with the corresponding immunosuppressant as monotherapy (HR = 0.51, 95% CI 0.36 to 0.71, *P* < .0001). Cox proportional hazards model for drug discontinuation throughout the follow‐up period yielded a lower hazard rate in the group of tacrolimus with lower‐dose steroids (HR = 0.56, 95% CI 0.36 to 0.87, *P* = .0101; Figure 4A). Post hoc analysis detected no significant difference in drug maintenance between this group (76.4%) and rituximab group (79.6%). The proportion of patients remaining on therapy was significantly higher for the reference group (66.8%) compared with lower‐dose steroids (44.4%), higher‐dose steroids (52.4%), azathioprine (45.2%), mycophenolate mofetil (40.5%) as monotherapy, or mycophenolate mofetil with lower‐dose steroids (50.8%) (HR = 2.27, *P* < .0001; HR = 1.44, *P* = .0145; HR = 1.91, *P* = .0079; HR = 1.53, *P* = .0446; respectively; Table 2, Figure 4A, and Figure 5A). The causes of therapy discontinuation differed between treatment groups. For the patients with combined therapy, MG relapses were the most common cause of drug discontinuation, followed by lower proportions of adverse events (Figure 4B, C, and D). For the patients with monotherapy, the most common cause of drug discontinuation in the group of higher‐dose steroids was higher percentage of adverse events (27.8%) (Figure 5B). Patients with lower‐dose steroids, azathioprine, and mycophenolate mofetil discontinued treatments due to MG relapses as the main reason (44.4%, 37.5%, 35.1%, respectively; Figure 5C, D, and E). Overall, lower drug discontinuation rates were found in the patients with tacrolimus or rituximab (Figure 5F and G).

The adverse events varied in each treatment group, but most were CTCAE grade 1. Hyperglycemia, liver dysfunction, and infection were the most adverse events in our cohort (Table S1).

A higher percentage of patients receiving mycophenolate mofetil with lower‐dose steroids (12.7%) and mycophenolate mofetil as monotherapy (8.1%) discontinued treatment due to pregnancy. The lowest discontinuation rates by pregnancy were recorded in patients on azathioprine with lower‐dose steroids (1.5%) and tacrolimus with lower‐dose steroids (1.8%).

### 3.7 Treatments for refractory cases

In this study population, 75 patients (7.0%) met the criteria for refractory generalized MG. 41 (54.7%) patients were anti‐AChR positive, 26 (34.7%) were anti‐MuSK positive, and 5 (6.7%) were anti‐LRP4 positive. These patients were administered efficacious regimens and achieved MMS eventually. Of all, 44 (58.7%) patients received rituximab treatment. 16 (21.3%) patients received tacrolimus and steroids treatment. 9 (12.0%) patients received 8 mg/kg/month intravenous tocilizumab treatment. Additionally, four cycles of subcutaneous bortezomib at a dosage of 1 mg/m^2^ of body surface area were used to deplete plasma cells in 6 patients. 2 patients had ever received belimumab due to concomitant systemic lupus erythematosus (SLE) but still experienced disease progression. They received rituximab in combination with bortezomib as add‐on steroids to attain the treatment goal.

## 4 DISCUSSION

Patients with generalized MG may be at a high risk of relapses if they discontinued immunotherapeutic treatments.^16^ In our cohorts, most patients who were in MMS generally remained stable for a longer period, 24.1% of all patients had relapses. Consistent with previous reports,^17^, ^18^ thymus hyperplasia without thymectomy and concomitant autoimmune diseases may promote MG relapse. The overall proportion of patients who maintained stable treatments decreased over the observation period. Steroid‐sparing azathioprine remained the most used immunosuppressant, but rituximab and steroid‐sparing tacrolimus were associated with superior efficacy as well as higher drug survival compared with other immunotherapeutic regimens. Most patients in our cohort who received rituximab were over 50 years old, and the efficacy of rituximab also contributed to reducing the risk of other concomitant immunotherapy in late‐onset MG patients.^19^

Clinical experience informed our cohort stratified usage of oral steroids in higher or lower dosages. Our data show that higher‐dose steroids (≥0.25 mg/kg) treatment significantly reduced risks of disease relapses compared to lower‐dose steroids (<0.25 mg/kg); however, this regimen recorded more adverse events prompting cessation of treatment. Use of a steroid‐sparing agent as add‐on with lower‐dose steroids regimes proved a viable alternative approach.^20^, ^21^ Our study indicated that steroid‐sparing tacrolimus showed superior efficacy as well as lower incidence of adverse events compared with azathioprine or mycophenolate mofetil. Indeed, consistent with prior reports, many patients could taper tacrolimus or even stop concomitant steroids.^22^, ^23^, ^24^ In light of limitations in treatment duration of mycophenolate mofetil as monotherapy or steroid‐sparing agent, patients who failed were more likely to switch to tacrolimus treatment. Overall, tacrolimus is tolerated better than azathioprine and mycophenolate mofetil in Chinese populations.

We identified that patients’ serostatus impacted treatment response. Anti‐MuSK positive patients had a significant higher risk of treatment failure with classical immunosuppressants compared with anti‐AChR positive patients. Although benefits from rituximab may be greater in anti‐MuSK positive patients, to control active disease than anti‐AChR positive patients,^25^, ^26^ rituximab was efficacious in maintaining remission in both cohorts of patients in this study.

The proportion of patients with refractory MG was lower than that of previous reports.^1^ One explanation was selection of patients who had achieved MMS. In addition, early usage of rituximab and tacrolimus may further account for this discrepancy, as efficacy in refractory cases has been demonstrated. Among patients with refractory generalized MG, rituximab remains the first choice of treatment. In patients with concomitant connective tissue diseases, especially systemic lupus erythematosus, disease activity was intractable without steroids and rituximab was usually used as add‐on. For the patients who also failed rituximab, several other treatments were tried in this study, including belimumab, tocilizumab, or bortezomib. Belimumab, a monoclonal antibody targeting soluble B‐cell activating factor, was used for patients with SLE in our cohort, but they suffered disease progression and did not achieve MMS, and its efficacy in generalized MG is not definite.^27^ Tocilizumab and bortezomib are shown to stabilize disease activity^28^, ^29^; however, the number of the patients receiving nonrecommended agents was small. The promising targets of antibody‐mediated effector functions may indicate the efficacy of Fc receptor inhibition,^30^ several trials blocking FcRn are on‐going, and future randomized control trials are warranted to assess long‐term safety and efficacy.

This observational cohort study is limited by a retrospective design. Selection bias due to heterogeneity of generalized myasthenia gravis was likely. Despite the overall large size of cohort, relatively small sizes of patients with rituximab or mycophenolate mofetil would have effects on outcomes compared to the other patients. Similarly, patients who received cyclosporine or methotrexate with daily lower‐dose steroids were also not enrolled due to small number. In addition, though initial therapy choice and the threshold for switching therapies were at the treating physicians’ discretion, patient‐oriented features such as price considerations were not deliberated in this study. In regard to change of therapy for efficacy reasons, our data support that such switches were prompted by objective evidence of disease relapses, manifested by significant changes in QMC scale. However, switching owing to safety or tolerability issues cannot be demonstrated in objective terms. Systemic recording of adverse events, as in prospective clinical trials, is lacking in the present study due to practical considerations of reporting. Lastly, newer immunotherapeutic agents, such as eculizumab, were not included in the study.

## 5 CONCLUSIONS

Patients who had attained MMS or better and then received stable treatments as maintenance demonstrate that regimens of rituximab as monotherapy and tacrolimus with lower‐dose steroids displayed improved efficacy and tolerability compared with other common immunotherapeutic regimens. Further studies are needed to clarify long‐term head‐to‐head comparative safety and effective outcomes of these different immunotherapies in large scale populations.

## CONFLICT OF INTEREST

The authors have no conflicts of interest to declare.

## AUTHOR CONTRIBUTIONS

F.‐D. S and CZ designed the study, collected, analyzed, and interpreted the data and drafted and revised the manuscript. PZ and CD did statistical analysis. BB, HY, LW, WL, R.‐SD, MZ, and LY collected and analyzed the data and revised the manuscript critically for intellectual content. Authors involved in drafting the text and figures were CZ, PZ, CD, and F.‐DS All authors approved the final version of the manuscript.

## Supporting information

Supinfo

## Data Availability

The datasets generated and analyzed during the current study are available from the corresponding author on reasonable requests.
