# Supplementary material for: Immunotherapy choice and maintenance for generalized myasthenia gravis in China
Source: CNS Neurosci Ther. 2020 Oct 26;26(12):1241–54. doi: 10.1111/cns.13468 (PMC7702233; doi:10.1111/cns.13468)
Supplement: Supplementary file 1 — Supinfo [file CNS-26-1241-s001.docx]

**Supplementary Files**

**Supplementary Figure 1.**


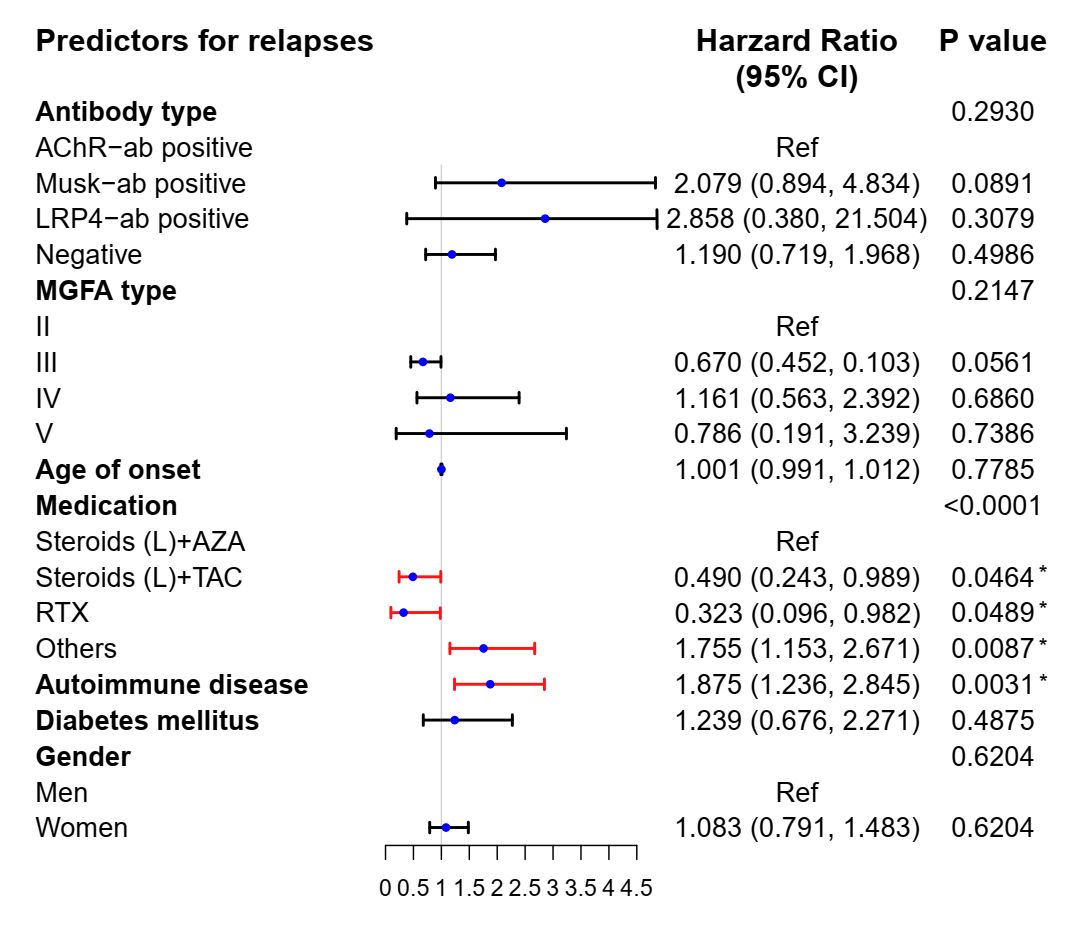


**Supplementary Figure 1.** HRs for predictors and treatments. HR and 95% CIs for clinical predictors of treatment response and different treatments (lower dose steroids with azathioprine as reference). AZA, azathioprine; TAC, tacrolimus; RTX, rituximab. * Represent statistically significant data (*P* < .05)

**Supplementary TABLE 1 Adverse events summarized in treatment groups**

|  | **Steroids (L)+AZA** | **Steroids (L)+MMF** | | **Steroids (L)+TAC** | **Steroids (L)** | **Steroids (H)** | | **AZA** | **MMF** | | **TAC** | **RTX** |
| --- | --- | --- | --- | --- | --- | --- | --- | --- | --- | --- | --- | --- |
| **Any** | 127 (47.4%) | 27 (42.9%) | 35 (31.8%) | | 41 (28.9%) | 102 (44.9%) | 63 (55.3%) | | 23 (62.6%) | 19 (29.7%) | | 13 (26.5%) |
| **Any leading to discontinuation of agent** | 25 (9.3%) | 6 (9.5%) | 11 (10.0%) | | 13 (9.2%) | 63 (27.8%) | 12 (11.5%) | | 6 (16.2%) | 11 (17.2%) | | 5 (10.2%) |
| **Adverse event reported in ≥ 10% of patients in either group, n (%)** | | | | | | | | | | | | |
| Hyperglycemia | 37 (13.8%) | 9 (14.3%) | 14 (12.7%) | | 27 (19.0%) | 101 (44.5%) | 2 (1.9%) | | 1 (2.7%) | 5 (7.8%) | | 0 |
| Abnormal liver function | 41 (15.3%) | 6 (9.5%) | 4 (3.6%) | | 4 (2.8%) | 7 (3.1%) | 42 (40.4%) | | 5 (13.5%) | 8 (12.5%) | | 1 (2.0%) |
| Infection^†^ | 29 (10.8%) | 5 (8.0%) | 8 (7.3%) | | 12 (8.5%) | 16 (7.0%) | 8 (7.7%) | | 4 (10.8%) | 9 (14.1%) | | 3 (6.1%) |
| Nausea and vomiting | 36 (13.4%) | 7 (11.1%) | 5 (4.5%) | | 2 (1.4%) | 17 (7.5%) | 13 (12.5%) | | 5 (13.5%) | 7 (10.9%) | | 1 (2.0%) |
| Anemia | 43(16.0%) | 4 (6.3%) | 4 (3.6%) | | 2 (1.4%) | 3 (1.3%) | 21 (20.2%) | | 3 (8.2%) | 3 (4.7%) | | 2 (4.1%) |
| Lymphopenia | 25 (9.3%) | 2 (3.2%) | 1 (0.9%) | | 0 | 0 | 33 (31.7%) | | 7 (18.9%) | 6 (9.4%) | | 4 (8.2%) |
| Flu-like syndromes | 5 (18.7%) | 5 (8.0%) | 8 (7.3%) | | 1 (0.7%) | 2 (0.9%) | 10 (9.6%) | | 3 (8.1%) | 4 (6.3%) | | 9 (18.4%) |
| Anaphylactic reaction | 2 (0.8%) | 1 (1.6%) | 2 (1.8%) | | 0 | 2 (0.9%) | 2 (1.9%) | | 1 (2.7%) | 3 (4.7%) | | 8 (16.3%) |
| **CTCAE grade, No (%)** | | | | | | | | | | | | |
| 1 | 103 (38.4%) | 23 (36.5%) | 51 (46.4%) | | 24 (16.9%) | 89 (39.2%) | 55 (52.9%) | | 20 (54.1%) | 17 (26.6%) | | 13 (26.5%) |
| 2 | 46 (17.2%) | 9 (14.3%) | 17 (15.5%) | | 11 (7.7%) | 34 (15.0%) | 31 (29.8%) | | 11 (29.7%) | 8 (12.5%) | | 6 (12.2%) |
| 3 | 4 (1.5%) | 1 (1.6%) | 2 (1.8%) | | 3 (2.1%) | 11 (4.8%) | 8 (7.7%) | | 3 (8.2%) | 2 (3.1%) | | 3 (6.1%) |
| 4 | 2 (0.8%) | 0 | 0 | | 0 | 6 (2.6%) | 2 (1.9%) | | 0 | 1 (1.6%) | | 0 |
| 5 | 0 | 0 | 0 | | 0 | 0 | 0 | | 0 | 0 | | 0 |

^†^ Infection indicated patients who had any forms of infection including respiratory tract infection, urinary tract infection or gastrointestinal infection. AZA, azathioprine; MMF, mycophenolate mofetil; RTX, rituximab; Steroids (H), higher-dose steroids; Steroids (L), lower-dose steroids; TAC, tacrolimus.
